# A metabolic engineering strategy for producing conjugated linoleic acids using the oleaginous yeast ***Yarrowia lipolytica***

**DOI:** 10.1007/s00253-017-8240-6

**Published:** 2017-03-29

**Authors:** Nabila Imatoukene, Jonathan Verbeke, Athanasios Beopoulos, Abdelghani Idrissi Taghki, Brigitte Thomasset, Claude-Olivier Sarde, Maurice Nonus, Jean-Marc Nicaud

**Affiliations:** 1 Micalis Institute, INRA, AgroParisTech, Université Paris-Saclay, 78350 Jouy-en-Josas, France; 2 Sorbonne Universités, EA 4297 TIMR, Université de Technologie de Compiègne (UTC), rue Personne de Roberval, F-60203 Compiègne, France; 3 Sorbonne Universités, FRE-CNRS 3580 GEC, Université de Technologie de Compiègne (UTC), rue Personne de Roberval, F-60203 Compiègne, France

**Keywords:** Conjugated linoleic acids, Oleaginous yeast, Lipid accumulation, *Yarrowia lipolytica*, Metabolic engineering

## Abstract

Conjugated linoleic acids (CLAs) have been found to have beneficial effects on human health when used as dietary supplements. However, their availability is limited because pure, chemistry-based production is expensive, and biology-based fermentation methods can only create small quantities. In an effort to enhance microbial production of CLAs, four genetically modified strains of the oleaginous yeast *Yarrowia lipolytica* were generated. These mutants presented various genetic modifications, including the elimination of β-oxidation (*pox1*-*6*∆), the inability to store lipids as triglycerides (*dga1*∆ *dga2*∆ *are1*∆ *lro1*∆*)*, and the overexpression of the *Y. lipolytica* ∆12-desaturase gene (Yl*FAD2*) under the control of the constitutive p*TEF* promoter. All strains received two copies of the p*TEF*-*oPAI* or p*POX*-*oPAI* expression cassettes; *PAI* encodes linoleic acid isomerase in *Propionibacterium acnes*. The strains were cultured in neosynthesis or bioconversion medium in flasks or a bioreactor. The strain combining the three modifications mentioned above showed the best results: when it was grown in neosynthesis medium in a flask, CLAs represented 6.5% of total fatty acids and in bioconversion medium in a bioreactor, and CLA content reached 302 mg/L. In a previous study, a CLA degradation rate of 117 mg/L/h was observed in bioconversion medium. Here, by eliminating β-oxidation, we achieved a much lower rate of 1.8 mg/L/h.

## Introduction

The conjugated linoleic acid (CLA) family is large. It contains 28 isomers of linoleic acid (LA; 18:2) that all feature a conjugated pair of double bonds that are separated by a single carbon bond. The conjugated double bonds can occur in several different positions, ranging from C6 to C14 (6, 8; 7, 9; 8, 10; 9, 11; 10, 12; 11, 13; or 12, 14). A variety of conformers exist (cis-cis, cis-trans, trans-cis, and trans-trans) (Banni 2002). The most common CLAs are c9t11-C18:2 (commonly known as rumenic acid) and t10c12-C18:2; they represent 90% of the CLA isomers found in milk fat. Interest in these compounds has grown ever since some CLAs were discovered to have physiological benefits (Lehnen et al. 2015), such as providing protection against atherosclerosis or cancer (Belury 2002; Crumb 2011). It has also been shown that t10c12-C18:2 can decrease body fat (De Deckere et al. 1999; Gavino et al. 2000). In the human diet, the major sources of CLAs are meat and dairy products obtained from ruminants (Chin et al. 1992; Shantha et al. 1994; McGuire et al. 1999). However, CLA production by ruminal bacteria is uncommon, and CLAs are scarce in foods. It is well known that linoleic acid isomerase converts LA into CLAs, and the linoleic acid isomerase found in *Propionibacterium acnes* (PAI) is the only enzyme with a characterized crystal structure (Liavonchanka et al. 2006). The enzyme uses free fatty acids as its only substrate. The *PAI* gene has been expressed in various CLA production systems, including those involving *E. coli*, *S. cerevisiae*, *Lactococcus lactis*, and tobacco and rice plants (Kishino et al. 2002; Ando et al. 2004; Hornung et al. 2005; Kohno-Murase et al. 2006; Rosberg-Cody et al. 2007).

*Yarrowia lipolytica* is an oleaginous yeast that can efficiently exploit hydrophobic substrates as its sole carbon and energy sources, and it has been shown to be a good model for lipid metabolism studies (Beopoulos et al. 2009b; Beopoulos and Nicaud 2012; Ledesma Amaro et al. 2016; Ledesma Amaro and Nicaud 2016). Past work has characterized the genes involved in the most important pathways in fatty acid degradation, triglyceride (TAG) synthesis, lipid remobilization, fatty acid transport, and fatty acid activation (Wang et al. 1999; Beopoulos et al. 2009a; Dulermo and Nicaud 2011; Beopoulos et al. 2012; Dulermo et al. 2014; Ledesma Amaro et al. 2016; Ledesma Amaro and Nicaud 2016). These pathways and their associated genes have been targets in studies focused on the development of obese *Y. lipolytica* strains, which can display lipid accumulation levels that exceed 80% of cell dry weight (CDW); the goal was to create an alternative strain for biofuel production (Beopoulos et al. 2008; Dulermo and Nicaud 2011). This species has also been used to produce rare fatty acids (Ledesma Amaro and Nicaud 2016) and has the advantage of being recognized as a safe-to-use microorganism (Groenewald et al. 2014). The most advanced process developed to date is the production of ώ3 fatty acids by DuPont-engineered *Y. lipolytica* strains (Zhu et al. 2015; Xie et al. 2015).

Two of the main target pathways for genetic manipulations are β-oxidation and TAG synthesis. Degradation of fatty acids in *Y. lipolytica* takes place exclusively via β-oxidation in peroxisomes. Six acyl-CoA oxidases (Aox1–6, encoded by the *POX1*–*POX6* genes, respectively) catalyze the first step. These acyl-CoA oxidases exhibit different activities and substrate specificities, as demonstrated by successive gene disruption (Wang et al. 1999). Two acyl-CoA oxidases, Aox2p and Aox3p, have high activity levels and display substrate specificity for long-chain and short-chain fatty acids, respectively. A strain in which all six genes were deleted (*pox1*-*6*∆) was unable to grow on lipid substrates and could not degrade fatty acids (Wang et al. 1999; Dulermo and Nicaud 2011).

Lipid storage depends on a key step in the synthesis of TAGs from diacylglycerol (DAG), which involves three DAG acytransferases (Dga1p, Dga2p, and Lro1p). TAGs can be synthesized by the acyl-CoA-dependent reaction catalyzed by Dga1p and Dga2p or by the acyl-CoA-independent pathway catalyzed by the phospholipid:diacylglycerol acyltransferase Lro1p. Moreover, *Y. lipolytica* possesses a sterol acyltransferase—acyl-CoA:cholesterol acyltransferase Are1p—that is not involved in TAG synthesis (Beopoulos et al. 2012). Strains from which all three DAG acyltransferases (Q3; *dga1*∆ *dga2*∆ *lro1*∆) or all four acyltransferases (Q4; *dga1*∆ *dga2*∆ *lro1*∆ *are1*∆) were deleted could not store TAGs and had high levels of free fatty acids in their cells (Beopoulos et al. 2009b). Strains that are incapable of carrying out fatty acid degradation and lipid synthesis (*pox1*-*6*∆ Q3 *fad2*∆; *pox1*-*6*∆ *dga1*∆ *dga2*∆ *lro1*∆ *are1*∆ *fad2*∆) have been used as chassis strains for ricinoleic acid production. The endogenous *FAD2*, *YALI0B10153g*, coding for the ∆12 desaturase catalyzing the transformation of oleic acid to linoleic acid, has also been deleted from these strains (Beopoulos et al. 2014).

Yeast-based CLA production was first explored using *S. cerevisiae* (Hornung et al. 2005) that expressed the *PAI* gene. By exploiting a codon-optimized version of the gene (*oPAI*), researchers were able to achieve an eightfold increase in CLA production. More recently, Zhang and colleagues (Zhang et al. 2012, 2013) evaluated CLA production in *Y. lipolytica*. In a first study (Zhang et al. 2012), which used the wild-type (WT) *PAI* gene, CLAs represented 0.2% of total fatty acids. However, yield was then improved by the insertion of multiple copies (up to 24) of *oPAI*: the percentage of t10c12-C18:2 reached 5.9% when neosynthesis medium was used. Unfortunately, the multi-copy strains were shown to be unstable. Subsequently, researchers combined a stronger promoter for *oPAI* expression (hp16d, a derivative of the hp4d promoter) with the coexpression of the Δ12 desaturase native to *Mortierella alpina* (under the control of hp4d and present in multiple copies). After the yeast strains were incubated for 38.5 h in a bioreactor, CLAs represented 10% of total fatty acids and 0.4% of CDW when neosynthesis medium (containing glucose) was used and 44% of total fatty acids and 30% of CDW when bioconversion medium (containing soybean oil) was used. However, the CLAs rapidly degraded (<10 h). When the yeast strains were incubated in flasks instead, CLA content did not surpass 45 mg/L (Zhang et al. 2013). This rapid degradation suggests that an optimal *Y. lipolytica* genetic background for CLA biosynthesis remains to be discovered.

In this study, we explored the outcome of genetic modifications to *Y. lipolytica* lipid metabolism pathways (β-oxidation and TAG synthesis) with a view to improving CLA production. We introduced two copies of o*PAI* into four strains with different genetic backgrounds and then analyzed CLA production under different conditions: in neosynthesis versus bioconversion medium and in flasks versus a bioreactor.

## Methods

### Yeast strains and culture conditions

**Fig. 1:**
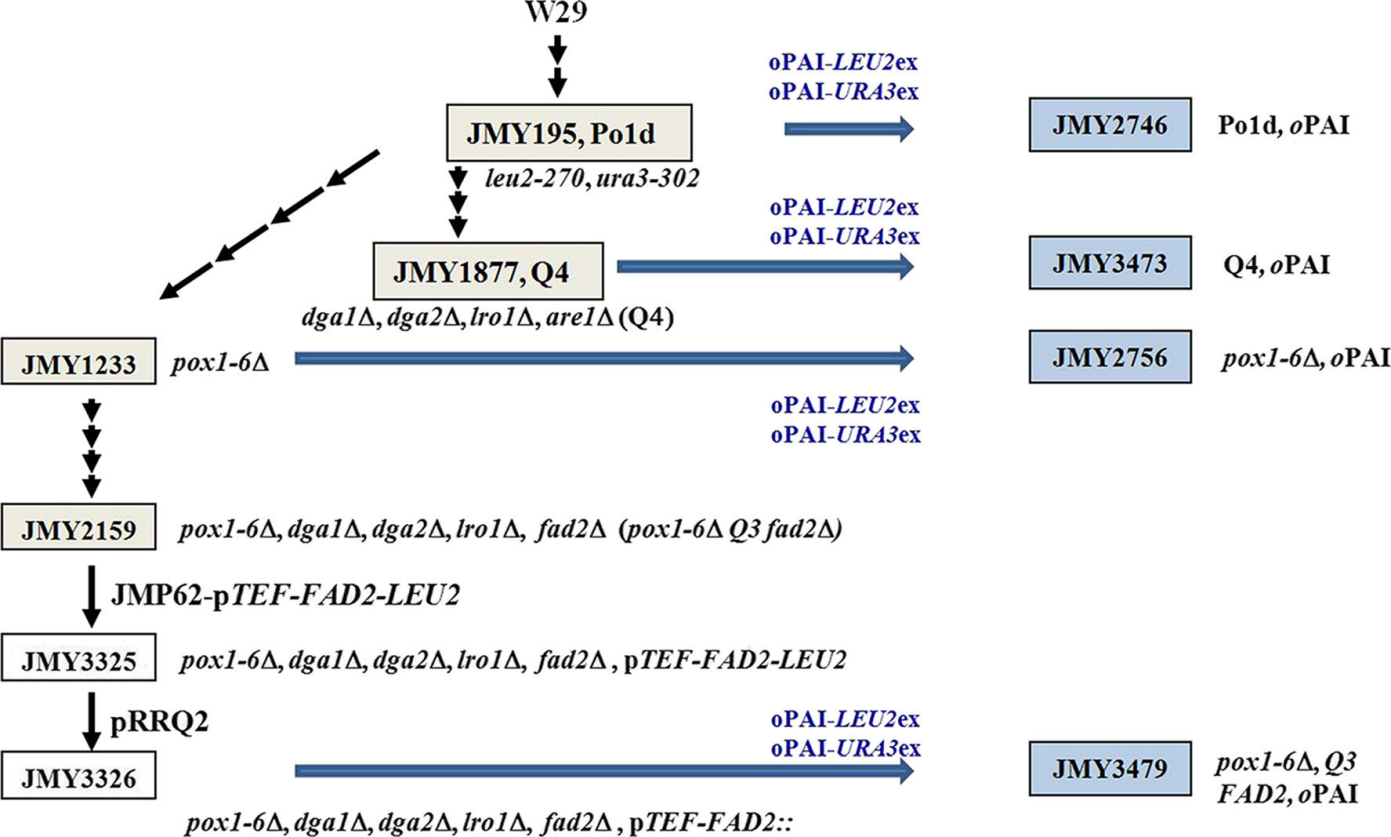
Schematic representation of strain construction. The auxotrophic strain Po1d (Leu−Ura−) was derived from WT strain W29. The construction of strains JMY195 (auxotrophic WT), JMY1233 (*pox1*-*6*Δ), JMY1877 (*dga1*Δ *dga2*Δ *lro1*Δ *are1*Δ [Q4]), and JMY2159 (*pox1*-*6*Δ *dga1*Δ *lro1*Δ *dga2*Δ *fad2*Δ) are described elsewhere (Barth and Gaillardin 1996; Beopoulos et al. 2008, 2012, 2014). The *gray boxes* contain intermediary strains, and the *blue boxes* contain study strains. Marker excision was performed with the replicative plasmid pUB4-CreI (pRRQ2). For *FAD2* and *oPAI* overexpression, coding genes were expressed under the p*TEF* or p*POX* promoter in the vector JMP62 containing either the *URA3ex* or *LEU2ex* excisable auxotrophic markers

The *Y. lipolytica* strains used were derived from the WT strain W29 (ATCC 20460). They are described in Table 1, and their construction is depicted in Fig. 1. The medium and growth conditions used for *E. coli* are described elsewhere (Sambrook et al. 1989), as are those for *Y. lipolytica* (Barth and Gaillardin 1996).

**Table 1:** Strains and plasmids used in this study

Strains	Plasmid, genotype	References
*E. coli*		
DH5a	80d*lac*Z∆m15, *rec*A1, *end*A1, *gyr*A96, *thi*-1, *hsd*R17 (r_k_-, m_k_+), *sup*E44, *rel*A1, *deo*R, ∆(*lac*ZYA*-arg*F)U169	Promega
JME547 (DH5a)	p*UB4*-*Cre1* (*Cre ARS68 Hyg in*)	This study
JME1514 (DH5a)	JMP62–p*POX*-*oPAI-URA3ex*	This study
JME1519 (DH5a)	JMP62-p*TEF*-*oPAI-URA3ex*	This study
JME1516 (DH5a)	JMP62–p*POX*-*oPAI-LEU2ex*	This study
JME1517 (DH5a)	JMP62-p*TEF*-*oPAI-LEU2ex*	This study
JME1346 (DH5a)	JMP62-p*TEF*-*FAD2-LEU2ex*	This study
*Y. lipolytica*		
JMY195 (Po1d)	MATA *ura3-302 leu2-270 xpr2-322*	Barth and Gaillardin (1996)
JMY1233 (*pox1*-*6*Δ)	Po1d, *pox1*-*6*Δ	Beopoulos et al. (2008)
JMY1877 (Q4)	Po1d, *dga1*Δ *dga2*Δ *lro1*Δ *are1*Δ	Beopoulos et al. (2012)
JMY2159 (*pox1*-*6*Δ, Q3, *fad2*Δ)	Po1d, *pox1*-*6*Δ *dga1*Δ *dga2*Δ *lro1*Δ *fad2*Δ	Beopoulos et al. (2014)
JMY3325	Po1d, *pox1* - *6*Δ *dga1*Δ *dga2*Δ *lro1*Δ *fad2*Δ, p*TEF*-*FAD2*-*LEU2*	This study
JMY3326	Po1d, *pox1*-*6*Δ *dga1*Δ *dga2*Δ *lro1*Δ *fad2*Δ, p*TEF*-*FAD2*	This study
JMY2746, CLIB 3036	Po1d; p*POX*-*oPAI-LEU2ex*, p*POX*-*oPAI-URA3ex*	This study
JMY2756, CLIB 3037	*pox1*-*6*∆; p*POX*-*oPAI-LEU2ex*, p*POX*-*oPAI-URA3ex*	This study
JMY3473, CLIB 3038	Q4; p*TEF*-*oPAI-LEU2ex*, p*TEF*-*oPAI-URA3ex*	This study
JMY3479, CLIB 3039	*pox1*-*6*∆, Q3, *fad2*∆, p*TEF*-*FAD2*; p*TEF*-*oPAI-LEU2ex*, p*TEF*-*oPAI-URA3ex*	This study

Strains were deposited at the French CIRM-Levures collection under CLIB number

(http://www6.inra.fr/cirm_eng/Yeasts)

Rich medium (YPD), minimal glucose medium (YNB), and minimal medium with leucine (YNBleu) or uracil (YNBura) were prepared as per Mlícková et al. (2004). Flask and bioreactor neosynthesis medium (YED_5_) contained 0.5% (*w*/*v*) yeast extract (Fisher Scientific, Illkirch Graffenstaden, France) and 5% (*w*/*v*) glucose (Cargill, Saint-Germain-en-Laye, France). The bioconversion media—linoleic acid (LA) flask medium (YNBD_1_-LA_2_) and soybean oil (SO) bioreactor medium (YNBD_1_-SO_2_)—contained 0.17% (*w*/*v*) yeast nitrogen base (without amino acids and ammonium sulfate: YNBw/w; Difco, Paris, France); 0.15% (*w*/*v*) yeast extract (Fisher Scientific, Illkirch Graffenstaden, France); 1% (*w*/*v*) glucose (Cargill, Saint-Germain-en-Laye, France); 0.5% (*w*/*v*) NH_4_Cl (VWR, Fontenay-Sous-Bois, France); 0.5 M phosphate buffer (pH 6.8); and either 2% (*v*/*v*) LA (67% purity; Sigma, Saint Louis, Missouri, USA) or 2% (*v*/*v*) SO (62% purity; Carrefour, France). The emulsion of LA and SO at 20% was sonicated for three cycles of 1 min on ice (fatty acid 10 mL, H_2_O 10 mL, Tween 80 0.05% (*v*/*v*)). The fatty acid composition of the LA was 4% C16:0, 2% C18:0, 30% C18:1, and 62% C18:2; that of the SO was 13% C16:0, 6% C18:0, 24% C18:1, and 57% C18:2.

Shake flask cultures were carried out in 250-mL baffled flasks containing 50 mL of each culture medium and incubated at 28 °C with stirring (160 rpm) for 72 h. The pH of the culture was adjusted to 6 for neosynthesis medium and 6.8 for bioconversion medium. Culture in 5-L bioreactor (TRYTONI, Pierre Guérin, Niort, France) was performed with a working volume of 3.5 L at 28 °C, aeration rate of 1 vvm, and agitation speed of 700 rpm.

### General genetic techniques

Genomic DNA was extracted from recombinant yeast clones as described elsewhere (Hoffman and Winston 1987) and used for PCR amplification. Amplified fragments were purified with the QIAgen Purification Kit (Qiagen, Hilden, Germany). Digested DNA fragments were subject to electrophoresis and recovered from 1% agarose gels using the QIAquick TM Gel Extraction Kit (Qiagen, Hilden, Germany). The restriction enzymes were obtained from Eurogentec SA (Liége, Belgium), and the DNA polymerase GO Taq came from Promega (Promega, Madison, WI, USA). Yeast cells were transformed using the lithium acetate method (Gaillardin et al. 1985).

### Plasmid construction and marker excision

Excision of the *LEU2ex* and *URA3ex* markers was performed with the replicative plasmid pRRQ2 (JME547) using the Cre-lox recombination system as described by Fickers et al. (2003). Genes of interest were placed under the control of either the constitutive *TEF* promoter or the OA-induced *POX* promoter. Briefly, the coding sequence was inserted between the *Bam*HI-*Avr*II restriction sites of the vector, which was derived from JMP62 and contained the p*TEF* or p*POX* promoter and the *LEU2*ex or *URA3*ex selective marker (Fig. 1). To change the selective marker in the vector, the *I-Sce*I cloning endonuclease site was used. Plasmids were linearized with *Not*I and the expression cassettes were purified after gel electrophoresis prior to transformation. Primers used for gene amplification and strain verification are described in Table 2.

**Table 2:** Primers used in this study

Primers	Sequence (5′→3′)	Restriction site(s)
Leu2sens	CGCTGTTGAGGCTGCCGTCAAGGAGTCCG	
Ura3sens	CGGCCAGCATGAGCAGACCTCTGGCCAG	
FAD2for*Bam*HI	CTCACGGATCCCACAATGGATTCGACCACGCAGACCAACACCG	*Bam*HI
FAD2rev*Avr*II	CCTAGCCTAGGCTACTTTTTAGAAGGCAGGCCGTCAGGAGC	*Avr*II
PAIfor*Bam*HI	CTGGATCCCACAATGTCTATTTCTAAGGACTCTCGAATCGCTATC	*Bam*HI
oPAIrev*Avr*II	GTCCCTAGGTTACACGAAGAATCGGGTGACCAGATC	*Avr*II

### Determination of biomass

Biomass was quantified using cell dry weight (CDW). The culture medium was centrifuged at 8000 rpm (772.6 g) for 10 min. The pellet was washed once with distilled water for neosynthesis medium and twice with BSA 0.5% and once with NaCl 0.9% for bioconversion culture medium, and its mass (i.e., weight) was determined after it was freeze-dried overnight.

### Lipid analysis

The lipids from 20 mg of freeze-dried cultured cells were directly converted into methyl esters, which were analyzed via gas chromatography (GC), as described in Browse et al. (1986). The GC analysis was performed using a GC-2010 Plus apparatus (Shimadzu, France) equipped with a flame ionization detector. A BPX 70G1203 column (30 m, 0.25 mm, 0.25 μm; SGE Analytical Science, Europe) was used to characterize the fatty acid methyl esters (FAMEs). They were identified via comparison to commercial standards (CLA, CLA methyl ester, FAME37, Supelco; Sigma, Saint Louis, Missouri), and their levels were quantified using an internal standard—100 μg of commercial C17:0 (Sigma, Saint Louis, Missouri, USA).

## Results

### Construction of recombinant yeast strains

Zhang and colleagues showed that CLA production could be enhanced by using codon-optimized *PAI*, the strong promoter hp16d, and the defective marker *ura3d4* (Zhang et al. 2012, 2013); the latter was developed in our laboratory for multiple copy integration. However, they also found that gene expression and CLA production were mutant dependent and that their strains were unstable (Zhang et al. 2012, 2013). In this study, we wanted to compare outcomes when different genetic backgrounds were used, which meant we could not exploit hp16d or the *ura3d4* system. However, we did use *oPAI*, expressed under either the strong p*TEF* or p*POX2* promoter; we introduced two copies using either the *URA3* or the *LEU2* marker (Table 1).

Four different genetic backgrounds were used: (1) JMY195 (the auxotrophic WT strain Po1d); (2) JMY1233 (*pox1*-*6*Δ), which has six disrupted acyl-CoA oxidase genes and is thus unable to degrade fatty acids via β-oxidation (Beopoulos et al. 2008); (3) JMY1877 (Q4), from which genes for four acyltransferases—Dga1p, Dga2p, Lro1p, and Are1p—were deleted and which was thus unable to synthesize TAGs (Beopoulos et al. 2012); and (4) JMY3326 (*pox1*-*6*Δ *dga1*Δ *dga2*Δ *lro1*Δ *fad2*Δ, p*TEF*-*FAD2*), which was derived from JMY2159 (*pox1*-*6*Δ, Q3, *fad2*Δ). In JMY2159 (*pox1*-*6*Δ, Q3, *fad2*Δ), genes related to β-oxidation (*pox1*-*6*Δ) and TAG synthesis (*dga1∆ dga2∆ lro1∆*), as well as the ∆12-desaturase gene (*FAD2*), were deleted (Beopoulos et al. 2008). To create JMY3326, JMY2159 was first transformed with a copy of the *Y. lipolytica FAD2* gene expressed under the p*TEF* promoter; this process gave rise to JMY3325 (Fig. 1, Table 1). Second, the *URA3* marker was rescued upon transformation with the replicative plasmid pUB4 as described in the “Methods” section, giving rise to JMY3326.

Two copies of *oPAI* were introduced into these four genetic backgrounds, giving rise to the prototroph strains JMY2746 (Po1d-*oPAI*), which represented the WT genotype; JMY2756 (*pox1*-*6*∆-*oPAI*), which could not perform β-oxidation; JMY3473 (Q4-*oPAI*), which could not synthesize TAGs; and JMY3479 (*pox1*-*6*∆-Q3-*FAD2*-*oPAI*), which could neither perform β-oxidation nor synthesize TAGs but which overexpressed ∆12 desaturase, the enzyme responsible for converting OA (18:1) to LA (18:2) (Fig. 1). Hereafter, JMY2746, JMY2756, JMY3473, and JMY3479 will be referred to as Po1d, *pox1*-*6*∆, Q4, and *pox1*-*6*∆-Q3-FAD2, respectively.

### Initial culturing conditions

The four study strains were first cultivated in flasks in either neosynthesis medium—where they were given 50 g/L glucose (YED_5_) as their carbon source—or in bioconversion medium—where they were given 20 g/L LA (YNBD_1_LA_2_) as their carbon source. Since some strains were incapable of β-oxidation (the *pox1*-*6*Δ genotype), 10 g/L of glucose was added to the bioconversion medium to allow growth.

### Lipid and CLA production dynamics depend on genetic background

#### Production in neosynthesis medium

**Fig. 2:**
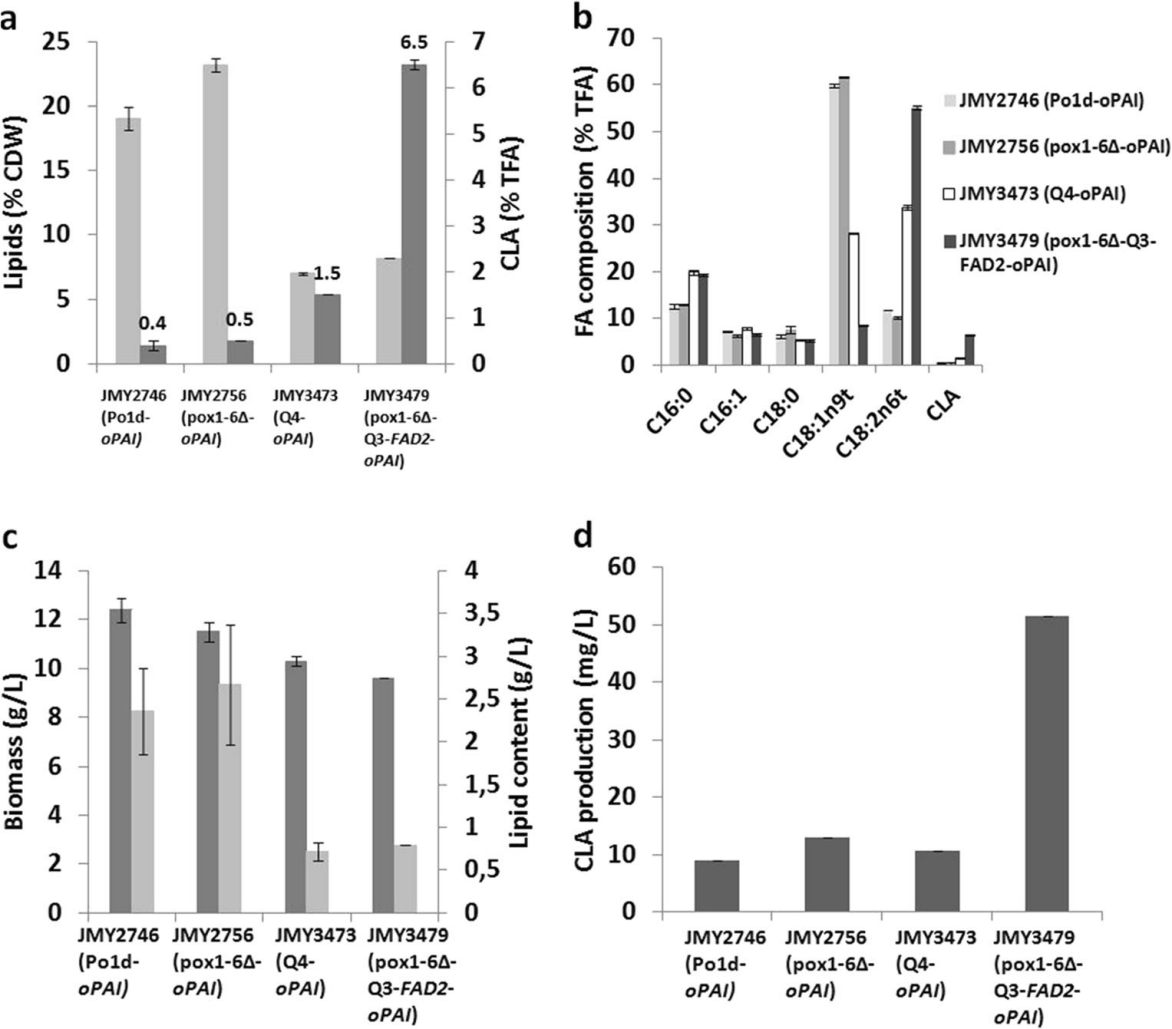
Strain production dynamics in neosynthesis medium in flasks. **a** Lipid production expressed as a percentage of CDW (*light gray*) and CLA production expressed as a percentage of total fatty acids (% TFA; *dark gray*). **b** Relative fatty acid composition (% TFA). **c** Biomass in terms of CDW (g/L; *dark gray*) and lipid content (g/L; *light gray*). **d** CLA production (mg/L). The results presented are the mean values ± SD for two independent biological replicates for the following strains: JMY2746 (Po1d-*oPAI*), JMY2756 (*pox1*-*6*Δ-*oPAI*), JMY3473 (Q4-*oPAI*), and JMY3479 (*pox1*-*6*Δ-Q3-*FAD2*-*oPAI*)

When yeasts were grown in the neosynthesis medium, lipid accumulation patterns varied according to genetic background (Fig. 2). First, we focused on the effects of eliminating β-oxidation. After 72 h of culture, in the Po1d-*oPAI* WT (JMY2746) and the *pox1*-*6*∆-*oPAI* strain (JMY2756), lipids accounted for 19.0 ± 0.9% and 23.2 ± 0.5% of CDW, respectively. In contrast, in the Q4-*oPAI* strain (JMY3473) and the *pox1*-*6*∆-Q3-*FAD2*-*oPAI* strain (JMY3479), lipids represented just 7.0 ± 0.1% and 8.0 ± 0.1% of CDW, respectively (Fig. 2a). As for CLA production, in the Po1d-*oPAI* WT (JMY2746) and the *pox1*-*6*∆-*oPAI* strain (JMY2756), CLAs accounted for 0.4 ± 0.1% and 0.5 ± 0.1% of total fatty acids, respectively. Thus, the removal of β-oxidation resulted in a 21% increase in total lipid production and a 25% increase in CLA production. However, in relative terms, CLA production remained rather limited.

We then looked at the effects of eliminating TAG synthesis, a drastic decrease in lipid content was observed in the relevant strains: 7.0% of CDW in the Q4-*oPAI* mutant (JMY3473) and 8.2% of CDW in the *pox1*-*6*∆-Q3-*FAD2*-*oPAI* mutant (JMY3479). CLAs accounted for 1.5 ± 0.1% and 6.5 ± 0.1% of total fatty acids, respectively. Thus, the elimination of TAG synthesis resulted in a major increase in CLA production: it was threefold greater in the Q4-*oPAI* mutant (JMY3473), but it was nearly 15-fold greater in the *pox1*-*6*∆-Q3-*FAD2*-*oPAI* mutant (JMY3479), which also could not perform β-oxidation and which overexpressed *FAD2*.

Fatty acid profiles also varied according to genetic background (Fig. 2b). The Po1d-*oPAI* WT (JMY2746) mainly accumulated OA (18:1), which represented about 60 ± 0.3% of total fatty acids; in contrast, this number was around 10% for LA (18:2). However, in the Q4-*oPAI* strain (JMY3473), OA and LA levels were similar (28 and 34%, respectively). A different pattern was seen in the *pox1*-*6*∆-Q3-*FAD2*-*oPAI* strain (JMY3479): LA accounted for 55% of total fatty acids, which meant there was more substrate for PAI. Indeed, in this strain, LA-to-OA ratio was about 6.5, while it was 1.5 in the Q4-oPAI strain (JMY3473) and just 0.2 in the other two strains. These numbers mean there was a 32.5-fold difference between the Po1d-*oPAI* WT (JMY2746) and the *pox1*-*6*∆-Q3-*FAD2*-*oPAI* mutant (JMY3479).

For the *pox1*-*6*∆-*oPAI* strain (JMY2756), we observed a small gain in lipid content that seemed to come at the expense of biomass production (Fig. 2c). As seen elsewhere (Wang et al. 1999), it would seem that the absence of β-oxidation affects growth and biomass production without affecting total lipid levels in this strain. Maximum CLA content (52 mg/L) was seen in the *pox1*-*6*∆-Q3-*FAD2*-*oPAI* strain (JMY3479) after 72 h of culture (Fig. 2d); the production rate was 0.8 mg/L/h. CLA content was slightly higher in the *pox1*-*6*∆-*oPAI* strain (JMY2756) than in either the Po1d-*oPAI* WT (JMY2746) or the Q4-*oPAI* strain (JMY3473).

### Production in bioconversion medium

**Fig. 3:**
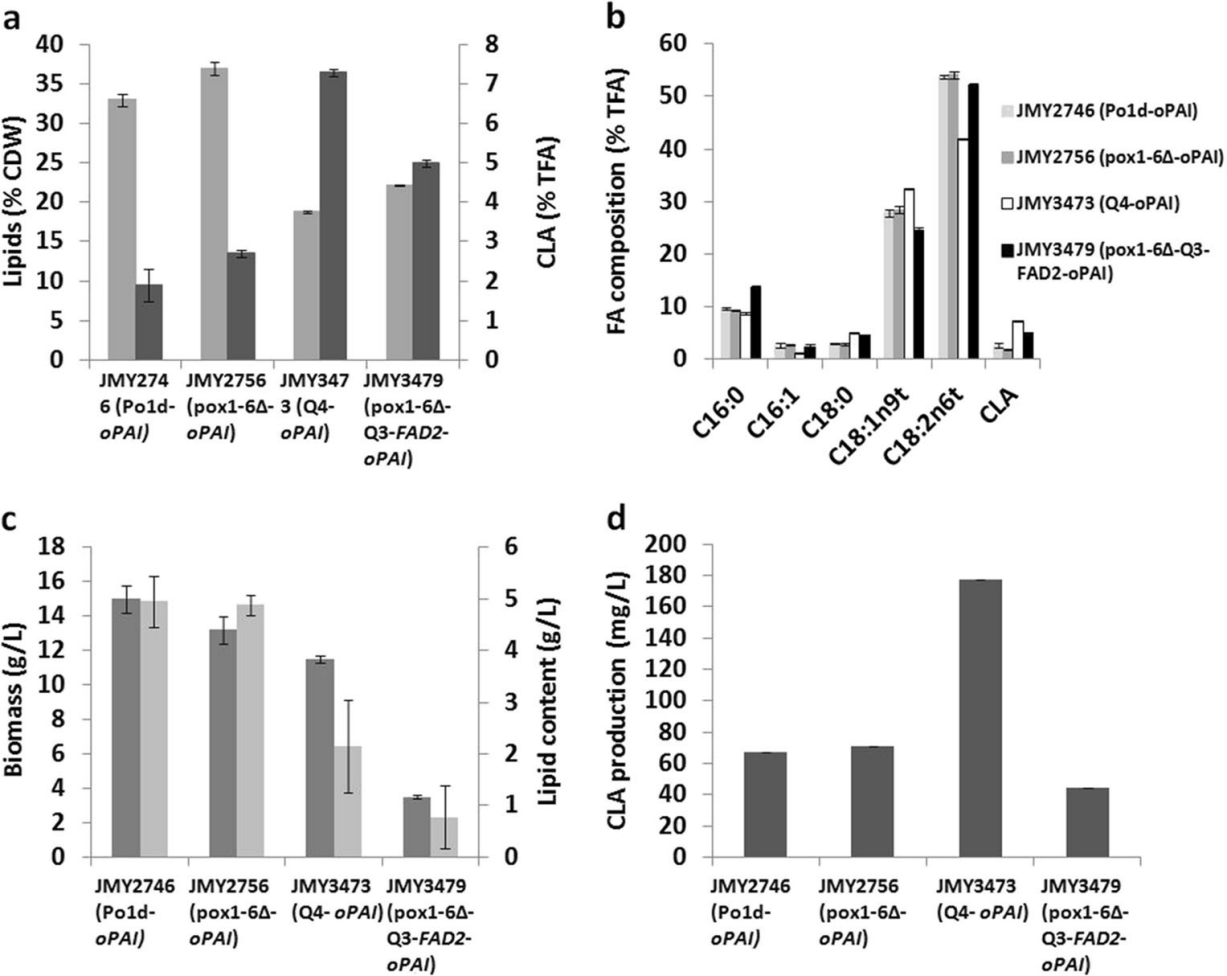
Strain production dynamics in bioconversion medium (LA) in flasks. **a** Lipid production expressed as a percentage of CDW (*light gray*) as well as CLA production expressed as a percentage of total fatty acids (% TFA; *dark gray*). **b** Relative fatty acid composition (% TFA). **c** Biomass in terms of CDW (g/L; *dark gray*) and lipid content (g/L; *light gray*). **d** CLA production (mg/L). The results presented are the mean values ± SD for two independent biological replicates for the following strains: JMY2746 (Po1d-*oPAI*), JMY2756 (*pox1*-*6*Δ-*oPAI*), JMY3473 (Q4-*oPAI*), and JMY3479 (*pox1*-*6*Δ-Q3 *FAD2*-*oPAI*)

When the yeast was cultured in linoleic acid bioconversion medium, there were clear differences in lipid production among strains. The Po1d-*oPAI* WT (JMY2746) and the *pox1*-*6*∆-*oPAI* strain (JMY2756) had significantly higher levels of lipid production than did the Q4-*oPAI* strain (JMY3473) or the *pox1*-*6*∆-Q3-*FAD2*-*oPAI* strain (JMY3479). Indeed, in the former two strains, lipids represented 33 ± 0.9% and 37 ± 0.6% of CDW, respectively, while in the latter two strains, lipids made up just 18.8 ± 0.2% and 22.2 ± 0.5% of CDW, respectively (Fig. 3a). After 72 h of culture, CLAs represented 1.9 ± 0.4%, 2.7 ± 0.1%, 7.3 ± 0.1%, and 5 ± 0.1% of total fatty acids in the Po1d-*oPAI* WT (JMY2746), the *pox1*-*6*∆-*oPAI* strain (JMY2756), the Q4-*oPAI* strain (JMY3473), and the *pox1*-*6*∆-Q3-*FAD2*-*oPAI* strain (JMY3479), respectively (Fig. 3a). Comparing the results for the *pox1*-*6*∆-*oPAI* strain (JMY2756) with those for the Po1d-*oPAI* WT (JMY2746), it is clear that the elimination of β-oxidation led to a 12% increase in lipid production and a 42% increase in CLA production. However, in relative terms, CLA production still remained low.

The strains incapable of synthesizing TAGs displayed a decrease in lipid content: 18.8 and 22.2% for the Q4-*oPAI* strain (JMY3473) and the *pox1*-*6*∆-Q3-*FAD2*-*oPAI* strain (JMY3479), respectively. However, their levels of CLA production were higher; CLAs represented 7.3 and 5% of total fatty acids, respectively. Thus, when the results were compared to those for the Po1d-*oPAI* WT (JMY2746), eliminating TAG synthesis resulted in a more than fourfold increase in CLA production in the Q4-*oPAI* strain (JMY3473) and an almost threefold increase in CLA production in the *pox1*-*6*∆-Q3-*FAD2*-*oPAI* strain (JMY3479).

All the strains contained higher percentages of LA (18:2) because of its inclusion in the medium (Fig. 3b). LA accounted for approximately 53 ± 0.5% of total fatty acids in the Po1d-*oPAI* WT (JMY2746), the *pox1*-*6*∆-*oPAI* strain (JMY2756), and the *pox1*-*6*∆-Q3-*FAD2*-*oPAI* strain (JMY3479) and OA was 26.9 ± 0.7%. In the Q4-*oPAI* strain (JMY3473), LA and OA, respectively, represented 42 ± 0.1% and 32 ± 0.1% of total fatty acids. The CLA-to-LA ratio in the Q4-*oPAI* strain (JMY3473) was twofold higher that in the *pox1*-*6*∆-Q3-*FAD2*-*oPAI* strain (JMY3479) (0.2 vs. 0.1) and fourfold higher than that in the Po1d-*oPAI* WT (JMY2746) or the *pox1*-*6*∆-*oPAI* strain (JMY2756) (0.2 vs. 0.05), indicating CLA production was the best in the Q4-*oPAI* strain (JMY3473), where CLAs made up 7.3% of total fatty acids.

When considering biomass and lipid content (Fig. 3c), a slight decrease in biomass production was observed for the *pox1*-*6*∆-*oPAI* strain (JMY2756), which meant its lipid content (about 4.8 g/L) was almost equivalent to that of the Po1d-*oPAI* WT (JMY2746). In the *pox1*-*6*∆-Q3-*FAD2*-*oPAI* strain (JMY3479), biomass, lipid content, and CLA content were all low. This strain also had a much slower growth rate than the other strains (data not shown). This strain displayed decreased viability after 48 h of culture (data not shown). Indeed, strains unable to degrade lipids via β-oxidation or store them as TAGs are more sensitive to free fatty acids, therefore affecting conversion of LA into CLAs. The LA thus remained in cells in the form of free fatty acids. Although the strain overexpressed ∆12 desaturase, its CLA production was low. In contrast, CLA content reached 177 mg/L in the Q4-*oPAI* strain (JMY3473) (Fig. 3d)—the maximum for the four strains—which translated to a production rate of 2.5 mg/L/h.

As compared to the neosynthesis medium, the bioconversion medium led to increases in lipid production of 73, 61, 168, and 170% for the Po1d-*oPAI* WT (JMY2746), the *pox1*-*6*∆-*oPAI* strain (JMY2756), the Q4-*oPAI* strain (JMY3473), and the *pox1*-*6*∆-Q3-*FAD2*-*oPAI* strain (JMY3479), respectively. CLA production increased by 375, 440, and 386% for the Po1d-*oPAI* WT (JMY2746), the *pox1*-*6*∆-*oPAI* strain (JMY2756), and the Q4-*oPAI* strain (JMY3473), but decreased by 30% for the *pox1*-*6*∆-Q3-*FAD2*-*oPAI* strain (JMY3479), suggesting the latter did not convert the LA in the medium into CLAs. It would have remained in the form of free fatty acids and could thus have had a toxic effect.

### Effects of ∆12 desaturase overexpression on CLA production in a bioreactor

**Fig. 4:**
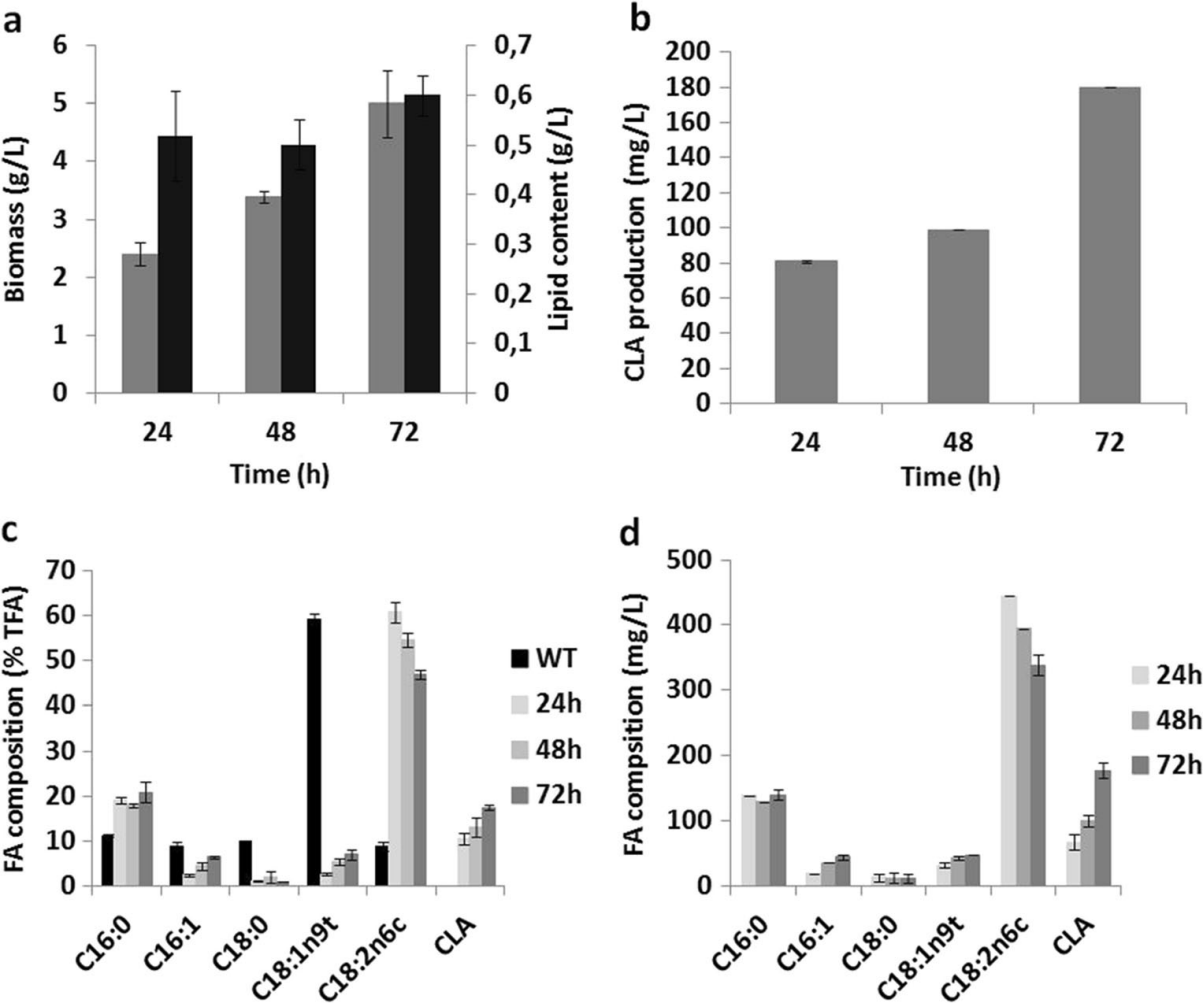
JMY3479 production dynamics in neosynthesis medium in a bioreactor. **a** Biomass in terms of CDW (g/L; in *gray*) and lipid content (g/L; in *black*). **b** CLA production (mg/L). **c** Fatty acid composition as percentage of total fatty acids (% TFA). **d** Fatty acid content (mg/L). JMY3479 (*pox1*-*6*Δ-Q3-*FAD2*-*oPAI*) displayed neither β-oxidation activity nor TAG synthesis but overexpressed Δ12 desaturase (Yl*FAD2*). The results presented are the mean values ± SD for two independent biological replicates

Further experimentation was carried out using the *pox1*-*6*∆-Q3-*FAD2*-*oPAI* strain (JMY3479). It was cultured in a bioreactor in neosynthesis medium for 72 h. Biomass, lipid content, and CLA content were determined every 24 h after inoculation. Lipid levels were analyzed using GC-FID. The results are shown in Fig. 4.

In the *pox1*-*6*∆-Q3-*FAD2*-*oPAI* strain (JMY3479), *Y. lipolytica*’s native Δ12 desaturase was disrupted (*fad2*Δ), and then a single copy was reintroduced under the control of the constitutive p*TEF* promoter, leading to the enzyme overexpression. This modification increased the availability of LA, which is the substrate for PAI. After 72 h of culture in neosynthesis medium in the bioreactor, the strain’s CLA content was six times greater than the CLA content observed in the other strains during growth in neosynthesis medium in flasks. Biomass increased over time, reaching 6 g/L after 72 h; a slight increase in lipid content was also observed (Fig. 4a). CLA content also increased over time, from 81 mg/L at 24 h to 180 mg/L at 72 h (Fig. 4b). When neosynthesis medium was used, bioreactor culturing led to a 50% increase in lipid production (8 ± 0.1% in flasks vs. 12 ± 0.1% in the bioreactor) and a more than 345% increase in CLA content (52 mg/L in flasks vs. 180 mg/L in the bioreactor). In addition, the overexpression of Δ12 desaturase had an effect on CLA production. Over time, there was a decrease in LA (Fig. 4c, d) and an increase in CLAs. OA was the predominant fatty acid (59 ± 1.3% of total fatty acids) found in the Po1d-*oPAI* WT (JMY2746), while LA made up 61 ± 2.2% of total fatty acids in the *pox1*-*6*∆-Q3-*FAD2*-*oPAI* strain (JMY3479) after 24 h of culture. The speed at which LA was converted to CLA (2.2 mg/L/h) was equal to the speed at which CLAs appeared (2.3 mg/L/h). Taken together, these results confirm that LA should be used as the main substrate for CLA production.

### Effects of soybean-oil medium on CLA production dynamics in a bioreactor

We also explored the possibility of using a vegetable oil to replace pure LA as the carbon source during the fermentation. More specifically, we conducted an experiment in which we used SO, which is rich in LA (57% of total fatty acids), instead of pure LA. We once again used the *pox1*-*6*∆-Q3-*FAD2*-*oPAI* strain (JMY3479): it was cultured in a 5-L bioreactor with YNBD-SO medium containing 2% (*v*/*v*) SO.

**Fig. 5:**
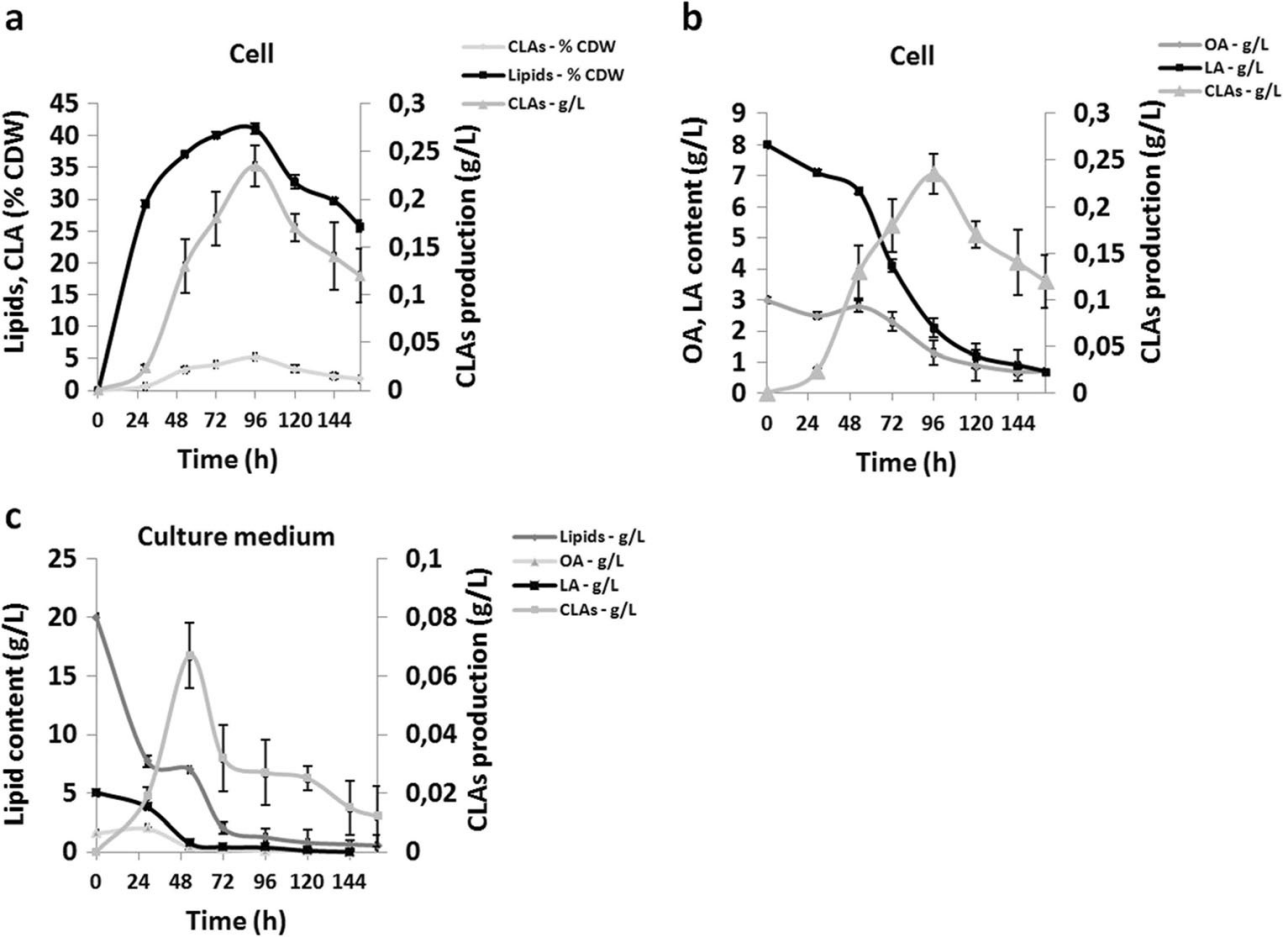
JMY3479 production dynamics in soybean-oil medium in a bioreactor. **a** Lipid and CLA production (% of CDW) and CLA production in cells (g/L). **b** Oleic acid (OA), linoleic acid (LA), and CLA production of cells (g/L). **c** Lipid, oleic acid (OA), linoleic acid (LA), and CLA production of the culture medium (g/L). JMY3479 (*pox1*-*6*Δ-Q3-*FAD2*-*oPAI*) displayed neither β-oxidation activity nor TAG synthesis but overexpressed Δ12 desaturase (Yl*FAD2*). All the results presented are the mean values ± SD for two independent biological replicates

After 96 h of culture, lipid and CLA levels had climbed, and they represented 40 and 5.2% (*w*/*w*) of CDW, respectively (Fig. 5a). Cellular levels of OA and LA increased and then decreased over time (Fig. 5b), indicating that OA was being converted to LA by Δ12 desaturase and then that LA was transformed into CLAs by PAI. After 96 h of culture, cellular CLA content reached 235 mg/L (Fig. 5b). The lipid content of the culture medium decreased from 20 g/L to zero over the first 96 h of culture (Fig. 5c), indicating that the SO was completely assimilated during fermentation (Fig. 5c). The CLA content of the culture medium built up, peaked at 53 h of culture, and then gradually declined (Fig. 5c). Over the first 96 h of culture, LA’s representation among the total fatty acids decreased from 58 to 47% in cells and from 56 to 34% in the culture medium (C18:2; Table 3). In contrast, over that same period, the relative percentage of CLAs increased from 0.2 to 6% in cells and from 0 to 3.4% in the culture medium. In absolute terms, CLA levels were at 302 mg/L after 96 h of culture in YNBD-SO medium in the bioreactor; this amount was six times higher than that obtained when the same strain was grown in flasks in neosynthesis medium (52 mg/L) or bioconversion medium (46 mg/L). It is also twice as large as the amount obtained when the strain was grown in a bioreactor in neosynthesis medium (180 mg/L).

**Table 3:** Fatty acid composition of the cellular and extracellular lipids produced by JMY3479 (*pox1*-*6*∆-Q3-*FAD2*-*oPAI*) in soybean-oil medium. Numbers represent the percentage of total fatty acids

Fatty acids	In cells			In culture medium	
	0 h	96 h	160 h	0 h	96 h
C16:0	10.9 ± 0.2	12.7 ± 0.6	15.6 ± 1.1	13.2 ± 0.7	24.2 ± 0.5
C16:1	0.02 ± 0.5	0.3 ± 1	0.45 ± 0.9	ND	7.1 ± 1
C18:0	3.4 ± 1.5	4.5 ± 0.7	7.6 ± 2	6.6 ± 1.7	10.7 ± 0.9
C18:1	25.1 ± 1.3	28.1 ± 2	33.5 ± 1.5	23.7 ± 0.7	16.2 ± 2
C18:2	58 ± 0.2	47.5 ± 1.4	34 ± 1	56.5 ± 1.8	34.5 ± 0.8
CLAs	0.2 ± 0.1	6 ± 0.2	5.4 ± 0.4	ND	3.4 ± 0.1

*ND* not detected

CLAs accumulated in cells at a rate of 3.1 mg/L/h between 29 and 96 h of culture (Fig. 5a); for the culture medium, the rate was 1.2 mg/L/h between 0 and 53 h (Fig. 5c). CLA degradation in cells occurred at a rate of 1.8 mg/L/h; it began at 36 h and continued until the end of culturing, at 160 h. In the culture medium, degradation was rapid between 53 and 72 h (1.8 mg/L/h) and then subsequently slowed significantly (0.2 mg/L/h) until the end of culturing, at 160 h.

## Discussion

The broad objective of this study was to metabolically engineer strains of the oleaginous yeast *Y. lipolytica* to express linoleic acid isomerase native to *Propionibacterium acnes* and thus efficiently produce CLAs. We examined CLA production in neosynthesis versus bioconversion medium and in flasks versus a bioreactor.

Our first step was to compare CLA production by four genetically modified strains grown in neosynthesis and bioconversion media (either containing just glucose for neosynthesis medium or glucose supplemented with LA as the carbon source for bioconversion medium). In neosynthesis medium, the *pox1*-*6*∆-*oPAI* strain (JMY2756) accumulated more lipids than did the Po1d-*oPAI* WT (JMY2746) (Fig. 2a), a pattern attributable to the elimination of β-oxidation in the former. This finding matches those of Dulermo and Nicaud (2011), who found that lipid and TAG accumulation were improved in a β-oxidation-deficient strain. Furthermore, Beopoulos et al. (2008) showed that, after 24 h of culture in glucose minimal medium, total fatty acid accumulation was over twice as high for a strain incapable of β-oxidation than for the wild-type strain. These results indicate that the six acyl-CoA oxidases play a significant role in lipid mobilization, as had been observed by Wang et al. (1999). Since the *pox1*-*6*∆-*oPAI* strain (JMY2756) was unable to degrade lipids, these lipids accumulated as TAGs or persisted as free or CoA-activated fatty acids; this fact may explain the low levels of CLA production in this strain. Lipid and TAG accumulation patterns were also determined for the Q4-*oPAI* strain (JMY3473) and the *pox1*-*6*∆-Q3-*FAD2*-*oPAI* strain (JMY3479). In contrast to the Po1d-*oPAI* WT (JMY2746) and the *pox1*-*6*∆-*oPAI* strain (JMY2756), their relative levels of lipid accumulation were just 7 and 8%, respectively.

These results show the important role of the four acyltransferases in the synthesis of storage lipids, which was also demonstrated in Beopoulos et al. (2012). Compared to other strains, the *pox1*-*6*∆-Q3-*FAD2*-*oPAI* strain (JMY3479), from which three acyltransferases and six acyl-CoA oxidases were eliminated and which overexpressed Δ12 desaturase, produced a relatively higher percentage of CLAs (6.5% of total fatty acids) after 72 h of culture. In this study, we introduced two copies of the *oPAI* gene and obtained a CLA production rate of 0.8 mg/L/h and a maximum CLA content value of 52 mg/L. In contrast, using eight copies of the *oPAI* gene, Zhang et al. (2013) obtained a production rate of 0.6 mg/L/h and a maximum content value of 45 mg/L (in YPD medium).

Lower CLA production was expected in strains with normal β-oxidation activity, such as the Po1d-*oPAI* WT (JMY2746) and the Q4-*oPAI* strain (JMY3473). However, it was surprising that it was also low in the *pox1*-*6*∆-*oPAI* strain (JMY2756). It is possible that the acyltransferases had a high affinity for OA and were extremely efficient at performing TAG acylation, which could have resulted in a pool of oleyl phospholipids that was too small or that remained inaccessible to the desaturase and isomerase. Regardless of the underlying mechanisms, in neosynthesis medium, CLA production was six times greater in the *pox1*-*6*∆-Q3-*FAD2*-*oPAI* strain (JMY3479) than in the other strains. This pattern results from the strain’s overexpression of Δ12 desaturase, which resulted in conversion of OA into LA, and its inability to degrade lipids. Consequently, LA became freely available to PAI: at 72 h of culture, LA represented 55% of total fatty acids (Fig. 2b). Previous work has found that *Y. lipolytica* grown in glucose medium for the de novo synthesis of fatty acids preferentially forms LA due to the overexpression of endogenous Δ12 desaturase (Meesters et al. 1996). In this study, when LA was present in the medium, all the strains accumulated large amounts of lipids (Fig. 3a). Lipid accumulation in the *pox1*-*6*∆-*oPAI* strain (JMY2756) was twice as high as in the Q4-*oPAI* strain (JMY3473) or the *pox1*-*6*∆-Q3-*FAD2*-*oPAI* strain (JMY3479). However, biomass and CLA content (Fig. 3c) was lower in the latter. This strain displayed decreased viability after 48 h of culture (data not shown which could have resulted from the toxic effects of free fatty acids that were present in higher concentrations in the cell, leading to cell lysis as observed previously) (Beopoulos et al. 2014, Dulermo et al. 2014, 2015). Dulermo and Nicaud (2011) showed that OA-induced cell death in *pox1*-*6*Δ and *gut2*Δ *pox1*-*6*Δ strains overexpressing GPD1 was due to the strains inability to export fatty acids. CLA production in medium containing LA was the greatest when the Q4-*oPAI* strain (JMY3473) was used, as it could not accumulate fatty acids as TAGs. It could, however, degrade free fatty acids, which suggests that it could have efficiently converted free LA into CLAs.

In this study, the potential bottleneck in CLA production was the intracellular concentration of LA. We found that, in the *pox1*-*6*∆-Q3-*FAD2*-*oPAI* strain (JMY3479), LA represented 61% of total fatty acids in cells, as compared to just 33% in the other strains. However, the relative percentage of CLAs was only slightly higher in this strain than in the others (6.5 vs. 1.5%; Fig. 2b). A possible explanation is that the LA produced by Δ12 desaturase is esterified and thus cannot serve as a substrate for PAI. Because PAI is soluble in the cytoplasm, it may compete with other cytosolic enzymes for free LA. These alternative enzymes could create different esterified forms of LA via various pathways, such as oxidation or elongation and thus ultimately restrict the availability of free LA for CLA production. Previous work has shown that expression of the Δ12-desaturase gene from *Mortierella alpina* in *Saccharomyces cerevisiae* led to reasonable levels of LA production (up to 25% of total fatty acids; Huang et al. 1999). When Zhang et al. (2013) used the Δ12-desaturase gene from *Mortierella alpina* expressed under the control of the hp16d promoter, the relative percentage of LA was 35.9%.Soybean oil, which is naturally rich in LA, was efficiently taken up by *Y. lipolytica* and then converted to CLAs (Fig. 5a). This observation suggests that exogenous LA, in contrast to de novo LA, is a good substrate for PAI (Fig. 5b). Furthermore, CLAs were detected in the growth medium (Fig. 5c), which could have resulted from the very high activity levels of intracellular lipases in *Y. lipolytica*: hydrolysis of the soybean oil could have occurred quickly, producing free fatty acids faster than they could be incorporated into TAGs or other forms of intracellular lipids. Because the *pox1*-*6*∆-Q3-*FAD2*-*oPAI* strain (JMY3479) could not accumulate lipids as TAGs, the free fatty acids could be secreted from the cells. This was previously reported for ricinoleic acid secretion of by Beopoulos et al. (2014) and by for fatty acid production by Ledesma et al. (2016). However, genes involved in fatty acid transport into the cell and genes involved in the secretion of fatty acid remains to be discovered.

We observed that CLA levels decreased after 53 h in the growth medium and after 96 h in the cells, which suggests that CLAs were degraded by auxiliary enzymes, forming intermediate compounds such as t2t4c6-trienoyl-CoA. Gurvitz et al. (2001) showed that t10c12-CLA degradation in *Saccharomyces cerevisiae* requires auxiliary enzymes. An alternative explanation could be the reuptake of the secreted fatty acid in the late stationary phase as described by Scharnewski et al. (2008). In cells, CLA degradation occurred at a rate of 1.8 mg/L/h. In the culture medium, the degradation rate was also 1.8 mg/L/h between 53 and 72 h of culture but then subsequently decreased to 0.2 mg/L/h. This pattern could indicate that the expression or activity of the main auxiliary enzymes drops after 72 h. Similar CLA degradation dynamics were observed by Zhang and colleagues. In their study, the degradation rate was 117 mg/L/h in cells and 95 mg/L/h in the growth medium (Zhang et al. 2013). This rate is framed by both the degradation of CLAs in the peroxisome via β-oxidation and the conversion of CLAs into OA by an auxiliary enzyme. This fact means that the elimination of β-oxidation could significantly decrease the CLA degradation rate.

In conclusion, this study has demonstrated the importance of acyltransferases in lipid accumulation and TAG production. Although the elimination of TAG synthesis improved CLA content slightly, lipid content remained low. When TAG synthesis was removed at the same time that Δ12 desaturase was overexpressed (in the *pox1*-*6*∆-Q3-*FAD2*-*oPAI* strain [JMY3479]), CLA content was maximal. Nonetheless, lipid content remained low when neosynthesis medium was used. In contrast, in bioconversion medium, CLA production was much greater in the Q4-*oPAI* strain (JMY3473), which could not synthesize TAGs.

Deletion of the β-oxidation pathway greatly reduced CLA degradation; indeed, the rate was even lower than that achieved in a study by Zhang et al. (2013). However, some degradation still occurred, probably due to the activity of an auxiliary enzyme. When the *pox1*-*6*∆-Q3-*FAD2*-*oPAI* strain (JMY3479) was cultured in the soybean-oil medium, its CLA production was similar to that of the strain containing 24 copies of the *oPAI* gene created by Zhang et al. (2013).

These results pave the way for further exploration of CLA production by *Y. lipolytica*: genetic modifications can be combined with the overexpression of the *oPAI* gene under a strong promoter and the deletion of auxiliary enzymes involved in CLA degradation.

## References

[CR1] Ando A, Ogawa J, Kishino S, Shimizu S (2004). Conjugated linoleic acid production from castor oil by *Lactobacillus plantarum* JCM 1551. Enzym Microb Technol.

[CR2] Banni S (2002). Conjugated linoleic acid metabolism. Curr Opin Lipidol.

[CR3] Barth G, Gaillardin C (1996) *Yarrowia lipolytica*. In: Wolf K (ed) Non conventional yeasts in biotechnology, vol 1. Springer, Berlin, pp 314–388

[CR4] Belury MA (2002). Recent advances in nutritional sciences inhibition of carcinogenesis by conjugated linoleic acid: potential mechanisms of action. J Nutr.

[CR5] Beopoulos A, Nicaud JM (2012). Yeast: a new oil producer?. OCL - OC Lipides.

[CR6] Beopoulos A, Mrozova Z, Thevenieau F, Le Dall MT, Hapala I, Papanikolaou S, Chardot T, Nicaud JM (2008). Control of lipid accumulation in the yeast *Yarrowia lipolytica*. Appl Environ Microbiol.

[CR7] Beopoulos A, Cescut J, Haddouche R, Uribelarrea JL, Molina-Jouve C, Nicaud JM (2009). *Yarrowia lipolytica* as a model for bio-oil production. Prog Lipid Res.

[CR8] Beopoulos A, Chardot T, Nicaud JM (2009). *Yarrowia lipolytica*: a model and a tool to understand the mechanisms implicated in lipid accumulation. Biochimie.

[CR9] Beopoulos A, Haddouche R, Kabran P, Dulermo T, Chardot T, Nicaud JM (2012). Identification and characterization of DGA2, an acyltransferase of the DGAT1 acyl-CoA:diacylglycerol acyltransferase family in the oleaginous yeast *Yarrowia lipolytica*. New insights into the storage lipid metabolism of oleaginous yeasts. Appl Microbiol Biotechnol.

[CR10] Beopoulos A, Verbeke J, Bordes F, Guicherd M, Bressy M, Marty A, Nicaud JM (2014). Metabolic engineering for ricinoleic acid production in the oleaginous yeast *Yarrowia lipolytica*. Appl Microbiol Biotechnol.

[CR11] Browse J, Mccourt PJ, Somerville CR (1986). Fatty acid composition of leaf lipids determined after combined digestion and fatty acid methyl ester formation from fresh tissue. Anal Biochem.

[CR12] Chin SF, Liu W, Storkson JM, Ha YL, Pariza MW (1992). Dietary sources of conjugated dienoic isomers of linoleic acid, a newly recognized class of anticarcinogens. J Food Compos Anal.

[CR13] Crumb DJ (2011). Conjugated linoleic acid (CLA)—an overview. Int J Appl Res Nat Prod.

[CR14] De Deckere EAM, Van Amelsvoort JMM, Mcneill GP, Jones P (1999). Effects of conjugated linoleic acid (CLA) isomers on lipid levels and peroxisome proliferation in the hamster. Br J Nutr.

[CR15] Dulermo T, Nicaud JM (2011) Involvement of the G3P shuttle and β-oxidation pathway in the control of TAG synthesis and lipid accumulation in *Yarrowia lipolytica*. Metab Eng 13:482–491. doi:10.1016/j.ymben.2011.05.002 10.1016/j.ymben.2011.05.002 21620992

[CR16] Dulermo R, Gamboa-Meléndez H, Dulermo T, Thevenieau F, Nicaud JM (2014). The fatty acid transport protein Fat1p is involved in the export of fatty acids from lipid bodies in *Yarrowia lipolytica*. FEMS Yeast Res.

[CR17] Dulermo R, Gamboa-Meléndez H, Ledesma-Amaro R, Thévenieau F, Nicaud JM (2015). Unraveling fatty acid transport and activation mechanisms in *Yarrowia lipolytica*. Biochim Biophys Acta - Mol Cell Biol Lipids.

[CR18] Fickers P, Le Dall M, Gaillardin C, Thonart P, Nicaud J (2003). New disruption cassettes for rapid gene disruption and marker rescue in the yeast *Yarrowia lipolytica*. J Microbiol Methods.

[CR19] Gaillardin C, Ribet AM, Heslot H (1985). Integrative transformation of the yeast *Yarrowia lipolytica*. Curr Genet.

[CR20] Gavino VC, Gavino G, Leblanc MJ, Tuchweber B (2000). An isomeric mixture of conjugated linoleic acids but not pure cis-9, trans-11-octadecadienoic acid affects body weight gain and plasma lipids in hamsters. J Nutr.

[CR21] Groenewald M, Boekhout T, Neuvéglise C, Gaillardin C, van Dijck PWM, Wyss M (2014). *Yarrowia lipolytica*: safety assessment of an oleaginous yeast with a great industrial potential. Crit Rev Microbiol.

[CR22] Gurvitz A, Hamilton B, Ruis H, Hartig A, Hiltunen JK (2001). Degradation of conjugated linoleic acid isomers in the yeast *Saccharomyces cerevisiae*. Biochim Biophys Acta.

[CR23] Hoffman CS, Winston F (1987). A ten-minute DNA preparation from yeast efficiently releases autonomous plasmids for transformation of *Escherichia coli*. Gene.

[CR24] Hornung E, Krueger C, Pernstich C, Gipmans M, Porzel A, Feussner I (2005). Production of (10E,12Z)-conjugated linoleic acid in yeast and tobacco seeds. Biochim Biophys Acta.

[CR25] Huang YS, Chaudhary S, Thurmond JM, Bobik EG, Yuan L, Chan GM, Kirchner SJ, Mukerji P, Knutzon DS (1999). Cloning of Delta 12-and Delta 6-desaturases from *Mortierella alpina* and recombinant production of gamma-linolenic acid in *Saccharomyces cerevisiae*. Lipids.

[CR26] Kishino S, Ogawa J, Ando A, Omura Y, Shimizu S (2002). Ricinoleic acid and castor oil as substrates for conjugated linoleic acid production by washed cells of *Lactobacillus plantarum*. Biosci Biotechnol Biochem.

[CR27] Kohno-Murase J, Iwabuchi M, Endo-Kasahara S, Sugita K, Ebinuma H, Imamura J (2006). Production of trans-10, cis-12 conjugated linoleic acid in rice. Transgenic Res.

[CR28] Ledesma Amaro R, Nicaud JM (2016). *Yarrowia lipolytica* as a biotechnological chassis to produce usual and unusual fatty acids. Prog Lipid Res.

[CR29] Ledesma Amaro R, Lazar Z, Rakicka M, Guo Z, Fouchard F, Coq AMCL, Nicaud JM (2016). Metabolic engineering of *Yarrowia lipolytica* to produce chemicals and fuels from xylose. Metab Eng.

[CR30] Lehnen TE, da Silva MR, Camacho A, Marcadenti A, Lehnen AM (2015). A review on effects of conjugated linoleic fatty acid (CLA) upon body composition and energetic metabolism. J Int Soc Sports Nut.

[CR31] Liavonchanka A, Hornung E, Feussner I, Rudolph MG (2006). Structure and mechanism of the *Propionibacterium acnes* polyunsaturated fatty acid isomerase. Proc Natl Acad Sci U S A.

[CR32] McGuire MK, McGuire MA, Ritzenthaler K, Shultz TD (1999). Dietary source and intakes of conjugated linoleic acid intake in humans. In: Yurawecz MP, Mossoba MM, Pariza MW, Nelson GJ (eds) Advances in conjugated linoleic acid research, vol 1. AOCS Press, Champaign, IL, pp 369–377

[CR33] Meesters PAEP, Huijberts GNM, Eggink G (1996). High-cell-density cultivation of the lipid accumulating yeast *Cryptococcus curvatus* using glycerol as a carbon source. Appl Microbiol Biotechnol.

[CR34] Mlícková K, Roux E, Athenstaedt K, Andrea S, Daum G, Chardot T, Nicaud J (2004). Lipid accumulation, lipid body formation, and acyl coenzyme A oxidases of the yeast *Yarrowia lipolytica*. Appl Environ Microbiol.

[CR35] Rosberg-Cody E, Johnson MC, Fitzgerald GF, Ross PR, Stanton C, Stanton CC (2007). Heterologous expression of linoleic acid isomerase from *Propionibacterium acnes* and anti-proliferative activity of recombinant trans-10, cis-12 conjugated linoleic acid. Microbiol.

[CR36] Sambrook J, Fritsch E, Maniatis T, Sambrook J, Fritsch EF, Maniatis T (1989). Molecular cloning: a laboratory manual.

[CR37] Scharnewski M, Pongdontri P, Mora G, Hoppert M, Fulda M (2008). Mutants of *Saccharomyces cerevisiae* deficient in acyl-CoA synthetases secrete fatty acids due to interrupted fatty acid recycling. FEBS J.

[CR38] Shantha N, Crum A, Decker E (1994). Evaluation of conjugated linoleic acid concentrations in cooked beef. J Agric Food Chem.

[CR39] Wang HJ, Le Dall MT, Waché Y, Laroche C, Belin JM, Gaillardin C, Nicaud JM (1999). Evaluation of acyl coenzyme A oxidase (Aox) isozyme function in the n- alkane-assimilating yeast *Yarrowia lipolytica*. J Bacteriol.

[CR40] Xie D, Jackson EN, Zhu Q (2015). Sustainable source of omega-3 eicosapentaenoic acid from metabolically engineered *Yarrowia lipolytica*: from fundamental research to commercial production. Appl Microbiol Biotechnol.

[CR41] Zhang B, Rong C, Chen H, Song Y, Zhang H, Chen W (2012) De novo synthesis of trans-10, cis-12 conjugated linoleic acid in oleaginous yeast *Yarrowia lipolytica*. Microb Cell Factories 11:51. doi:10.1186/1475-2859-11-51 10.1186/1475-2859-11-51 PMC3390286 22545818

[CR42] Zhang B, Chen H, Li M, Gu Z, Song Y, Ratledge C, Chen YQ, Zhang H, Chen W (2013). Genetic engineering of *Yarrowia lipolytica* for enhanced production of trans-10, cis-12 conjugated linoleic acid. Microb Cell Factories.

[CR43] Zhu Q, Jackson EN, Betenbaugh MJ, Bentley WE (2015). Metabolic engineering of *Yarrowia lipolytica* for industrial applications. Curr Opin Biotechnol.

